# Medical practitioners’ perceptions of adverse event reporting for rare diseases in South Africa

**DOI:** 10.4102/hsag.v31i0.3540

**Published:** 2026-10-16

**Authors:** Leith F. Kwaan, Helen L. Malherbe, Colleen Aldous, David Blackbeard

**Affiliations:** 1 Department of Clinical Medicine, Nelson R. Mandela School of Medicine, College of Health Sciences, University of KwaZulu-Natal, Durban, South Africa; 2 Centre for Human Metabolomics, Desmond Tutu School of Medicine, Faculty of Health Sciences, North-West University, Potchefstroom, South Africa; 3 Department of Clinical Psychology, Grey’s Hospital, Pietermaritzburg, South Africa; 4 Department of Psychiatry, Nelson R. Mandela School of Medicine, College of Health Sciences, University of KwaZulu-Natal, Durban, South Africa

**Keywords:** qualitative study, reflexive thematic analysis, medical practitioner perceptions, adverse event reporting, rare diseases

## Abstract

**Background:**

Adverse events (AEs) are underreported. In rare diseases (RDs), this is compounded by small patient populations, complex pathology and limited treatments. Most related research is from developed countries using survey methods.

**Aim:**

This qualitative study aimed to expand on existing knowledge by exploring perceptions of medical practitioners (MPs) managing RDs regarding AE reporting.

**Setting:**

The study took place in the South African public and private healthcare sectors.

**Methods:**

A qualitative, exploratory and interpretive design was employed. Individual interviews were conducted with 12 purposively sampled MPs, exploring their perceptions and experiences of AE reporting, and relevant barriers and facilitators. Transcripts were analysed using reflexive thematic analysis.

**Results:**

Three themes were generated: (1) Trust in healthcare systems, colleagues and doctor–patient interaction impacted reporting. (2) Reporting AEs for medicines with delayed local launch was perceived to contribute insignificantly to safety profiles. Participants’ stewardship of limited treatment options caused reluctance to report AEs and caution around direct patient reporting. (3) Risk tolerance for treatment gain, including off-label use, was perceived to be balanced by medicine safety knowledge.

**Conclusion:**

Pharmacists were trusted for AE reporting. Medical practitioners’ reluctance to report AEs associated with singular treatment options was grounded in concern regarding treatment access. Caution around direct patient reporting was driven by a reluctance to jeopardise treatment access as a result of potentially irrelevant patient-reported events. Risks associated with medicines are mitigated by knowledge generated through AE reporting.

**Contribution:**

Issues surrounding trust relationships, resources and practice risk were identified as underlying AE underreporting by MPs. Proposed solutions included pharmacist involvement and improved medicine supply.

## Introduction

Pharmacovigilance practice in the post-market setting relies largely on spontaneous adverse event (AE) reporting (Bogolubova, Padayachee & Schellack 2018:1; Pal et al. 2013:75). Spontaneous AE reporting refers to unsolicited communication describing AEs by healthcare professionals or consumers that does not derive from a study (South African Health Products Regulatory Authority [SAHPRA] 2026:10). Adverse event reporting generates real-world data on the safety of medicines (Pal et al. 2013:75). Globally, AEs are underreported, with particularly low rates in developing countries, especially in Africa (Aagaard et al. 2012:1175–1177; Bogolubova et al. 2018:1; Pal et al. 2013:79). Among other factors, AE underreporting by medical practitioners (MPs) is attributed to limited knowledge of pharmacovigilance, complacency with respect to medicine safety, confidentiality concerns, time constraints, difficult or onerous reporting processes and fear of repercussion (Biagi et al. 2013:242; García-Abeijon et al. 2023:627, 631; Güner & Ekmekci 2019:17).

Extensive published research on this topic has been conducted through survey questionnaires directed at respondents in Europe (Biagi et al. 2013:238; Dittrich et al. 2026:110–112; Güner & Ekmekci 2019:14; Varallo et al. 2014:740–741). However, this method may be inadequate to capture the depth and complexity of MPs’ perceptions on the topic. Potentially unfulfilled reporting obligations make this a sensitive topic. Questionnaires are therefore subject to social desirability bias, in which respondents may underreport socially undesirable activities and/or over-report socially acceptable behaviour (Krumpal 2013:2028). To overcome this bias, a qualitative method was used in this study, incorporating individual interviews to produce transcripts that were thematically analysed (Braun & Clarke 2024:610). This approach expanded the discussion of the topic to explore the perceptions and experiences of MPs, particularly those treating rare diseases (RDs).

According to a panel of experts led by Rare Diseases International and the World Health Organization (WHO), an RD is a medical condition affecting 1 in 2000 people or less in any WHO-defined region (Wang et al. 2024:3). This definition has been adopted widely in South Africa (SA) (Pazaropoulos-Koot, Mahomed & Pienaar 2025:16). With a range of aetiologies, RDs: (1) can manifest at birth or later in life, predominantly in childhood, (2) are often life-threatening, (3) are usually chronic and progressive or degenerative and (4) can present with various lifelong disabilities (Wang et al. 2024:4). Medicines that are developed to treat RDs are designated as Orphan Medicines in many settings (Michaeli et al. 2024:982). Small RD patient populations and complex disease characteristics mean that Orphan Medicines may be brought to market with limited safety evidence (Michaeli et al. 2024:985). This makes post-market surveillance an important data source for generating safety profiles. Despite progress in medicine development, most RDs still lack an approved treatment (Jonker et al. 2024:1). Many RD treatments are costly (Kwaan, Aldous & Malherbe 2026:5), which further limits access, especially in a resource-constrained setting like SA. Limited treatment options for RDs may influence MPs’ perspectives and AE reporting decisions.

This study aimed to produce an integrated account of MPs’ experiences and perceptions of AE reporting for RDs in SA. The objective was to gain a meaningful understanding of barriers and facilitators of AE reporting and to identify potential strategies for improving reporting rates. The research question was: ‘What are the perceptions and experiences of MPs regarding AEs, their consequences and the barriers or facilitators of effective AE reporting in the context of RDs?’

## Research methods and design

### Setting

The study focused on AE reporting in routine medical practice, with exploration of the impact of the RD context on MPs’ perceptions. Virtually conducted interviews facilitated MPs’ participation while in their chosen physical environment. A mix of participants from urban and rural settings was intended; however, this was difficult to achieve, as most MPs who agreed to participate were in urban areas. This was likely because of the naturally higher density of medical specialists in large cities than in rural areas. Medical practitioners from all provinces were invited to participate, with successful recruitment from Gauteng, KwaZulu-Natal, Mpumalanga, Northern Cape and Western Cape.

To effectively answer the research question and to inform strategies to improve AE reporting, MPs were included from a range of settings in which people living with RDs may access care. The focus was on medical specialists because of routine RD patient referrals. Participants were recruited from the public and private healthcare sectors. A balanced mix of practice settings reflected the highly specialised nature of the participants and included specialist private practices, private hospitals and public district, tertiary and central hospitals.

### Design

A qualitative, exploratory design was used in this study as there were no preconceived expectations regarding the findings. Perceptions of AE reporting held by RD treaters have not been studied. Existing literature regarding MPs’ perceptions is largely based on survey questionnaires (Biagi et al. 2013:238; Dittrich et al. 2026:110–112; Güner & Ekmekci 2019:14; Varallo et al. 2014:740–741). These do not provide an in-depth exploration of underlying meaning or meaningfulness to MPs. Therefore, an explorative, interpretive design was selected to understand MPs’ AE reporting behaviour and its drivers and barriers. A descriptive approach was applied to practical aspects of participant responses; however, an interpretative analytical method was used to explore latent concepts that were meaningful to participants. Individual semi-structured interviews were analysed inductively with Reflexive Thematic Analysis (RTA) (Braun & Clarke 2024:609–610). Interview transcripts were coded with an emphasis on ‘latent’ (underlying meaning) over ‘semantic’ (surface meaning) codes. Themes were developed and refined in an iterative process. Reflexive thematic analysis is an interpretive method (Braun & Clarke 2004:609), so researcher reflexivity was applied in the context of a constructionist epistemology and an experiential orientation to the data.

The constructionist epistemology enabled co-creation of meaning and meaningfulness by researchers and participants through the coding process. The experiential orientation to the data placed emphasis on the perceptions of the participants. The inductive coding approach was data-driven, reflecting participants’ underlying perceptions, assumptions and ideas that informed meaningfulness.

### Study population and sampling strategy

The study population included South African MPs with experience in RDs. The RD context required the inclusion of several highly qualified medical specialists. Six paediatricians were included because of their key role in diagnosing and managing RDs. Five of them had an additional subspecialty, maintaining diversity among participants. Purposive sampling was used to invite MPs with maximum diversity in gender, race, practice setting, region and specialisation. Male and female MPs were invited from across SA, spanning all provinces and both public and private sectors, with the aim of ensuring representation across the four population groups commonly used in demographic reporting in the country (black African, white [European descent], Indian and coloured). Inclusion criteria were: (1) registered and practising as an MP in SA, (2) current or recent experience with RDs and (3) willingness and availability to participate in the research.

Thirty-nine MPs were invited to participate via: (1) invitation of MPs presenting at RD conferences (*n* = 4), (2) ‘cold call’ invitation of MPs identified through online searches (*n* = 24) or (3) invitation of referred MPs (*n* = 11). Some invited MPs were known to the authors, but this was limited to professional acquaintance. Bias in participant selection was avoided by including the first MPs who agreed to participate. Recruitment continued through the interview phase until information sufficiency was reached to answer the research question. Information sufficiency was independently confirmed by both researchers, based on the participants’ unique and common insights. The information power of the sample was confirmed using the five-element model by Malterud, Siersma and Guassora (2016:1754), including: (1) narrow study aim, (2) high participant specificity, (3) clear theoretical framework, (4) rich dialogue quality and (5) in-depth analysis strategy.

Participants received a summary of the study design and provided informed consent prior to being interviewed.

### Data collection

The study took place in SA from April to September 2025. Individual semi-structured interviews were used, consistent with an interpretive qualitative paradigm, allowing flexibility to explore participants’ views on potentially sensitive issues. The primary researcher (LK), a pharmacist, conducted all interviews; this shared professional background facilitated rapport and informed reflexive engagement during data collection. Interviews were conducted individually to support open discussion, providing deeper insight than survey methods and minimising potential constraints associated with group settings. This approach enabled in-depth exploration of participants’ perspectives, with meaning co-constructed through the interaction between interviewer and participant and carried forward into the iterative analysis of the transcripts.

Interviews were conducted via recorded videoconference using Microsoft Teams® (Microsoft Corp., Redmond, WA, United States) or telephone call, according to participant preference. Twelve initial interviews occurred, followed by six member-reflection interviews with participants who had agreed to further participation. This limited follow-up was intended to confirm appropriate interpretation of participants’ insights and further exploration of certain issues, without unnecessary burden on all participants. Semi-structured interviews were guided by the principles described by Brinkmann and Kvale (2015:21–22, 51). An interview guide was used, with six open-ended questions and 10 optional probing questions (refer to Online Appendix 1). This guide was systematically developed and tested using Castillo-Montoya’s Interview Protocol Refinement Framework, following four steps: (1) alignment of interview questions with the research question, (2) constructing an inquiry-based conversation, (3) receiving feedback on the interview protocol and (4) piloting the interview protocol (Castillo-Montoya 2016:812).

Interview audio files were transcribed using the built-in transcription function of Microsoft Teams® or the Apple iPhone® 11 (Apple Inc., Cupertino, CA, United States). These were reviewed against the original recordings by the primary researcher and refined to ensure accuracy. To maintain anonymity, participants were assigned a codename, used throughout the transcript. All other identifiers such as names, places and/or medicine trade names were redacted prior to analysis.

Researcher insights and initial impressions were noted during and directly after each interview. The primary researcher conducted all interviews and transcriptions, resulting in a high level of familiarity with the dataset, which informed reflexive, iterative coding and analysis.

### Data analysis

Interview transcripts were analysed using the six phases of RTA (Braun & Clarke 2024: Supplementary Material), specifically: (1) familiarisation with the data, (2) latent coding, (3) generating themes, (4) reviewing themes, (5) defining and naming themes and (6) producing the report. Familiarisation was conducted independently by both researchers. All other phases were conducted by the primary researcher (LK), in consultation and collaboration with the co-researcher (DB).

The primary researcher’s professional and epistemological reflexivity provided a unique perspective on this topic. The professional background as a pharmacist and relative proximity to participants informed the research process and were considered reflexively during data collection and analysis, alongside the study’s interpretive epistemological orientation. The primary researcher engaged in reflexivity throughout the analytical process, recognising their role in shaping data interpretation. An inductive coding approach, without a preconceived framework, was applied to allow themes to be derived from participants’ accounts, in line with the research aim.

### Trustworthiness

Measures of trustworthiness were used to ensure credibility, transferability, dependability and confirmability. The Interview Protocol Refinement Framework resulted in a dependable interview guide for intentional data collection. Recruiting until information sufficiency ensured appropriate sampling and representation among participants. Credibility was achieved through the privacy of individual interviews and the opportunity to engage freely using open-ended questions. The rapport during interviews further established credibility and trust, promoting candid and honest discussion. Confirmability was ensured through verification of observations in member-reflection interviews. As interviewer, transcriber and initial coder, the primary researcher (LK) had close familiarity with the data, supporting nuanced interpretation. Credibility and dependability were further supported by DB’s independently conducted RTA familiarisation step and contribution to coding. In creating themes, the RTA approach was systematically applied, according to predetermined methodological steps. The analytical output was evaluated against established qualitative research reporting standards and the specific RTA Reporting Guidelines by Braun and Clarke (2024: Supplementary Material) to support transparency and rigour. These methods were selected to produce a trustworthy record of the research.

The rich and nuanced account of participants’ insights contributed to transferability beyond this specific context. This mitigates the study limitation that participants were located mostly in urban areas in highly populated provinces. Participants were included from both public and private healthcare sectors, and some practised in both sectors. In the public sector, RD patients from rural hospitals would usually be referred to tertiary and central institutions generally located in urban areas. This referral mechanism ensured that the study’s participants were representative of those who treat a wide range of rural and urban patients in SA or similar settings.

### Ethical considerations

Full ethical approval was granted by the Biomedical Research Ethics Committee of the University of KwaZulu-Natal on 18 November 2024. The ethics approval number is BREC/00007656/2024. Interaction with participants was conducted in accordance with the ethical standards of the institutional research committee and with the 1964 Declaration of Helsinki and its later amendments.

Written informed consent was obtained from all individual participants using a consent form approved by the ethics committee, prior to the interviews. Participants were advised of their right to withdraw from the study and the subsequent action to be taken upon their withdrawal. This included, for post-interview withdrawal, the exclusion of their data from analysis. There were no participant withdrawals from the study. Participants were informed of the intention to publish the study findings, with no objections raised.

Privacy and confidentiality measures were communicated prior to obtaining informed consent. This included the researcher’s use of a private interview space and the responsibility of the participant for their own interview environment, accounting for their personal privacy considerations. To maintain participant confidentiality, all transcripts were de-identified (redacted) prior to analysis. In addition, medical specialisation has not been reported per participant (excluded from Table 1) to avoid participant identification through common demographics such as race, gender, province and specialisation.

**TABLE 1:** Participants’ demographic characteristics.

Codename	Gender	Race	Province	Practice setting	Interviews
MP-01	Male	Black African person	KwaZulu-Natal	Public	Initial; Member-reflection
MP-02	Male	White person	Northern Cape	Private	Initial; Member-reflection
MP-03	Female	Indian person	KwaZulu-Natal	Private	Initial
MP-04	Female	White person	Gauteng	Private	Initial; Member-reflection
MP-05	Male	White person	Western Cape	Public	Initial
MP-06	Female	Coloured person	KwaZulu-Natal	Public	Initial; Member-reflection
MP-07	Female	Black African person	Mpumalanga	Public; Private	Initial
MP-08	Female	White person	Western Cape	Public	Initial
MP-09	Female	White person	Gauteng	Private	Initial
MP-10	Male	Coloured person	Western Cape	Private	Initial
MP-11	Female	Black African person	Gauteng	Public; Private	Initial; Member-reflection
MP-12	Male	Indian person	KwaZulu-Natal	Public	Initial; Member-reflection

MP, medical practitioner.

To ensure data security, all interview files were stored on an unlinked personal device equipped with up-to-date Microsoft Windows^®^ 11 security features, including anti-virus and firewall protection.

## Results

Twelve participants were interviewed, as presented in Table 1. The purposive sample included a gender distribution of 5:7 male:female and a diverse racial distribution. Most participants were from the urbanised regions of Gauteng, KwaZulu-Natal and the Western Cape, reflecting the geographic spread of medical specialists across SA. They were equally distributed between public and private healthcare sectors. A broad range of medical specialities were represented, including dermatology, endocrinology, general surgery, gynaecology, haematology, immunology, nephrology, neurology (two participants), oncology, paediatrics (six participants), pulmonology and rheumatology. All participants were practising MPs involved in the management of RDs.

Initial interviews ranged from 15 min to 29 min (mean and median of 22 min), with follow-up interviews of 8 min – 20 min (mean 14 min; median 13 min). Interview duration was guided by participants’ availability and the depth of discussion, recognising their professional commitments. Participants’ communications were time-efficient but thoughtful, producing deep content and meaning that was interpreted and presented in three interrelated themes, each having two sub-themes, as depicted in Figure 1.

**FIGURE 1:**
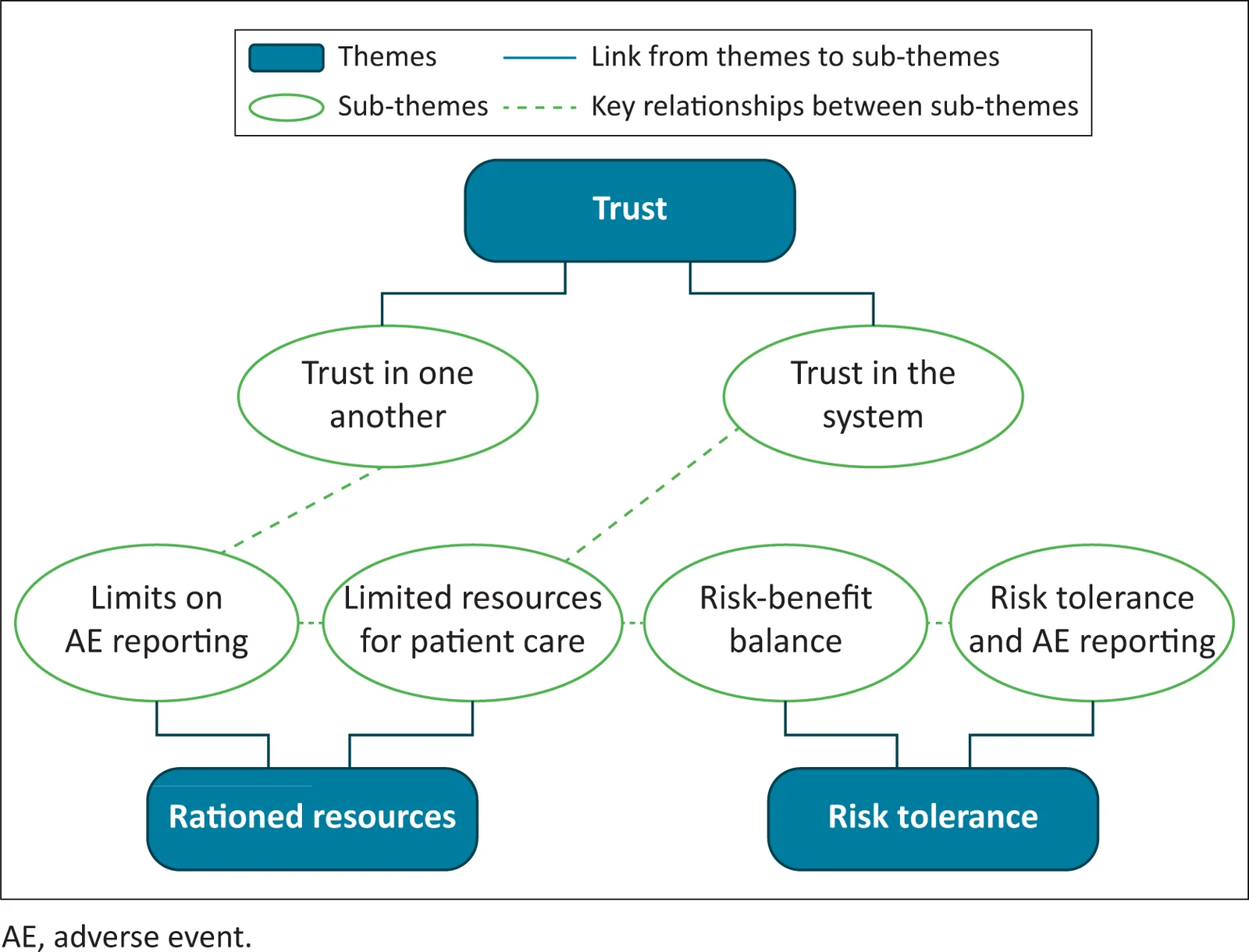
Map of themes and sub-themes generated through reflexive thematic analysis of semi-structured interview transcripts, showing key relationships between sub-themes.

### Theme 1: Trust

#### Sub-theme 1.1: Trust in one another

Participants perceived that they provided treatment and care for their patients within a trusted pharmaceutical context. This perception was rooted in their trust in the regulatory authority and established formulary committees that approve medicines based on evidence regarding safety, quality and efficacy. Some felt that, upon registration, safety of medicines should be fully established in all age groups and patient populations, including persons living with an RD. Others acknowledged the challenges in acquiring pre-market safety data in rare or vulnerable populations, recognising the need for off-label use in paediatric and RD settings. Participants also accepted the use of unregistered medicines in clinical trials, despite limited safety evidence.

One participant explained:

‘It’s a “caught-between-a-rock-and-a-hard-place” situation because if you have nothing else to give your patient, like as opposed to giving them fresh air, would you consider using this drug that may or may not be as rigorously tested?’ (MP-03, female, private sector)

Participants displayed genuine empathy for persons living with an RD. They were aware of the trust their patients placed both in them and in the medicines they prescribed. This seemed to foster a strong sense of responsibility for patient care, including communicating medicine risks to patients and advocating for their safety. Unintentional miscommunication by patients was cited as a barrier to AE reporting, as a result of inaccurate medical histories confounding MPs’ causality assessment. However, participants also described instances in which information was intentionally withheld by patients because of fear of judgment by the MP. A participant working in a public health setting stated:

‘They feel like […] you’re going to be angry with them or judge them, and that [*could not be*] further from the truth. All we want is to get a clear picture so that you know what to do, what to expect.’ (MP-06, female, public sector)

Patient advocacy organisations (PAOs) were perceived to offer value in terms of levelling the possible doctor–patient power differential by educating patients regarding their rights and responsibilities. This is especially important for rural patients, who were described as mistakenly regarding MPs as ‘demigods who know everything’ (MP-01, male, public sector). However, concerns were raised about PAOs’ potential involvement in the AE reporting process. Patient advocacy organisations were seen to have a potential conflict of interest arising from their funding sources or because of their agendas that might not always align with those of MPs.

In contrast, participants expressed a high level of trust for pharmacists, who were viewed as key resources in connecting MPs with medicine manufacturers for AE reporting. One participant explained:

‘I’m closer with the pharmacist than I am with the manufacturer. […] I think the pharmacist is a […] beautiful link between us, as clinicians, [*and*] the manufacturer.’ (MP-07, female, public and private sector)

Participants valued the role of pharmacists in several aspects of AE management. These included: (1) prevention, through identification of prescription errors; (2) identification, through critical evaluation of prescription changes; (3) evaluation, including assessment of drug interactions and pharmacological mechanisms of AEs; (4) reporting, specifically process-related tasks and centralising communication channels; (5) feedback, through participation in multidisciplinary forums; and (6) recording, as custodians of collated AE data at an institutional level. The medical referral system, often employed in AE management, involves several stakeholders who might report an AE. Participants indicated they may opt not to report, assuming a colleague had done so. By centralising the AE reporting process to the pharmacist, it was felt that the risk of report omission or duplication within a transdisciplinary medical team may be reduced and so improve the AE data submitted to pharmacovigilance surveillance systems.

Effective doctor–pharmacist collaboration was seen to mitigate risk incurred by MPs in their prescribing. However, tension was raised as a potential barrier in specific instances where some MPs may perceive that their prescriptions were negatively evaluated by pharmacists. Open communication and effective medical management were highlighted as keys to overcoming this challenge.

#### Sub-theme 1.2: Trust in the system

Supportive institutional structures also featured in discussions regarding the price of truth-telling with respect to AEs. Trust in management was raised by MPs as a facilitator of truthful and effective AE reporting. Repercussions or punitive measures were perceived to discourage honest AE reporting. This was highlighted with respect to medication error, which was perceived to be more likely in RDs as a result of limited familiarity in treating them. According to a Medical Manager interviewed:

‘It is a traumatic experience to realise that the AE happened because of your negligence. It’s important to support the clinician. So, if management is not supportive, I think the person is unlikely to report it.’ (MP-01, male, public sector)

There was awareness of litigation risk. While potential litigation was raised as a barrier to AE reporting, it was also cited as a motivator to perform due diligence in response to an AE. A participant managing a private practice explained:

‘It’s probably important […] from a medicolegal aspect […], because if you’ve done the right reporting and […] you get sued, you can say “But we did it, we managed it, we’ve reported it, we’ve done everything according to the books.”’ (MP-02, male, private sector)

In general, participants were in favour of truth-telling in all circumstances, as they were regarding open communication. However, they were candid about their perceptions regarding potential self-preservation behaviour among MPs in the context of AE reporting.

### Theme 2: Rationed resources – Care amid constraint

#### Sub-theme 2.1: Limits on adverse event reporting

Participants in this study work in resource-constrained settings, especially with respect to RDs. Some participants expressed frustration with the performance of the regulatory authority, with lengthy delays in medicine registration and complex routes of achieving medicine access and reporting AEs. The perceived delay in the local launch of novel medicines discouraged AE reporting. With lagging access to new products, there was a perception that AE reporting would not meaningfully contribute to post-market data already collected. One participant explained:

‘I don’t think we are […] on the cutting edge of medicine research, we’re mostly on the receiving end and that work’s been done. By the time we get it, with all our delays, the rest of the world has had it for 10 years.’ (MP-04, female, private sector)

The AE reporting process was perceived to be onerous, offering little or no direct benefit to MPs or patients. Participants consistently reported prioritising patient care (including treating AEs) over AE reporting. However, they did value clinical information regarding medicines and acknowledged the contribution of AE reports in this regard. This was seen as especially pertinent for underrepresented patient groups, and participants acknowledged the value in collecting data (including AEs) in an African population, despite delayed medicine access.

#### Sub-theme 2.2: Limited resources for patient care

Participants consistently steered the discussion toward the challenge of limited treatment options, especially with respect to RDs. This led to some electing not to report AEs associated with medicines for which there were limited or no alternatives. The risk of losing a treatment option was a foregrounded concern of the participants and a contributor to their decision-making. This reluctance to report was explained by a participant who worked frequently with RDs:

‘So, if I’m going to condemn this one with an AE that is not life-threatening, what alternatives do I have? Is it that the patient can live with this AE? Or is it so terrible that we need to stop? Those are the options that I have to weigh, because now if I’m condemning this one and I don’t have an alternative, what is my plan?’ (MP-11, female, public and private sector)

In some scenarios, participants perceived that the benefit to their patient outweighed the risk related to the observed AE. This perspective appeared to be consistently applied to act in the best interest of the patient by preserving their treatment. Treatment preservation seemed to outweigh their principled belief in the value of AE reporting as a contributor to knowledge regarding medicine safety profiles. The duty to advocate for patients was applied universally by participants, who felt that all patients deserved equitable access to care, including safe treatment options. These participants and their patients routinely face health inequality, both geographically and in terms of RDs. Limited resources create an ethical dilemma, as described by a public sector participant:

‘And the difficulty comes with the costing, and they are always told that “You’re busy fighting for one child and we could be treating 500 children,” […] but there’s also one parent who’s counting on you to help their one child.’ (MP-06, female, public sector)

Off-label use and repurposing of medicines were suggested as key means to improve treatment access. However, participants expressed disappointment with rigid bureaucratic policies and formularies that limit these practices. They were reluctant to engage with onerous bureaucratic processes but were willing to do so in applying for medicine access on a named patient basis. In contrast, the perceived lack of direct benefit from AE reporting, thought to be similarly onerous, provided no motivation for them to engage with the process.

The participants’ inclination toward treatment preservation was expressed in their concerns regarding direct patient reporting of AEs. They acknowledged patients’ right to report AEs but felt that patients lacked the expertise to accurately assign causality, especially in polypharmacy, and considering lifestyle or dietary factors. One participant illustrated this using an example:

‘We’ve got patients with polypharmacy. […] They’re on three agents. Now they have a drug reaction to one of them. Now they are going to go and report to [*the regulator*] what?’ (MP-06, female, public sector)

Participants were therefore concerned about spurious and potentially duplicate AE reports submitted by patients without MP involvement. They felt this could jeopardise the development of accurate post-market medicine safety profiles by contributing irrelevant or misleading data into pharmacovigilance systems. Ultimately, they believed that inappropriate AE reporting could lead to medicine withdrawal from the market.

### Theme 3: Risk tolerance for treatment gain

#### Sub-theme 3.1: Risk–benefit balance

There was a perception among participants that more aggressive treatment approaches carried a greater degree of risk for patients. Medical practitioners treating RDs might be managing a life-threatening condition with limited treatment options. In this context, there was an appreciation of newer, targeted therapies, contrasted with an acknowledgement of the associated risks, some of which could be life-threatening AEs. One participant noted:

‘The AEs are becoming more prominent and it’s more part of my discussion with a patient than what it used to be because the risks are actually greater.’ (MP-09, female, private sector)

Strong clinical data on these medicines reassures prescribers regarding appropriate use and was identified as a risk minimisation measure for MPs. These data, some of which are generated through AE reporting, equip MPs to inform patients regarding medicine risks, supporting evidence-based medicine and patient involvement in decisions regarding their care. Medicine knowledge generated through AE reporting was also seen to enable MPs to best position therapies to maximise patient benefit. For example, identifying second-line treatment strategies, exit therapy or preferred options for ageing or pregnant patients.

#### Sub-theme 3.2: Risk tolerance and adverse event reporting

Participants described how serious AEs were tolerated by both MPs and patients in the context of a life-saving treatment. This dilemma (risk of AE versus life-saving treatment benefit) seems to present a conflict for MPs who have limited options. Another conflict that seemed to underpin MPs’ decisions whether to report an AE was the requirement on them to continue prescribing a causative medicine despite AEs, as a result of the lack of alternatives. In RDs, participants explained how they might opt to continue to treat through an AE if the benefit of treatment outweighed the risk, for example:

‘A patient may come to me and have a drug reaction, but it’s not life-threatening, and it’s far more important for the patient to [*be treated*] on the drug than for me to withdraw. So, there are times when we treat through.’ (MP-06, female, public sector)

In these circumstances, participants cited different mechanisms used to assist patients. They highlighted the role of PAOs to support patients in managing AEs associated with life-saving medicines that could not be withdrawn.

Novel medicines were highlighted as important candidates for AE reporting to support evidence-based medicine and ongoing risk-benefit analysis. Participants described MPs’ navigation of their own risk incurred as they used new medicines. In RDs, MPs might prescribe medicines with limited familiarity. Participants identified that patient risk was reduced as MPs gained experience using novel medicines and prescribing them in RDs. A participant in private practice articulated the process of familiarisation:

‘The difficulty […] is getting to have a personal feel on the new drugs […] and what to expect, […] once you’re brave enough to use it.’ (MP-10, male, private sector)

Participants identified colleague support as an important risk minimisation measure, however, this tended to be limited for RDs:

‘With the RDs, again, you stand alone. So that’s often a difficult problem.’ (MP-10, male, private sector)

Regardless of the clinical circumstance, all participants displayed a strong commitment to providing the best possible patient care and a willingness to learn and consult, in order to minimise risks incurred by patients while obtaining maximum benefit.

## Discussion

Experiential semi-structured interviews were used to generate data that was analysed using RTA. This generated knowledge and deepened insights into MPs’ perceptions regarding AE reporting. The impact of the RD context on their reporting behaviour was also explored. The concept of trust was foundational to participants’ perceptions. Rationed or limited resources constrained treatment and care, affecting AE reporting. Themes involving trust and limited resources led participants into discussion regarding risk-benefit analysis, a key principle in pharmacovigilance (SAHPRA 2022:5).

### The role of trust in adverse event reporting

The role of trust in AE reporting underpinned several aspects of participant perceptions. The two areas of trust were the relationships between MPs and other healthcare stakeholders, and trust in the healthcare system (including management structures and pharmacovigilance systems).

Participants indicated that they implicitly trusted the safety of registered medicines. They were also willing to use unregistered medicines in clinical trials for RDs. This reflected their trust in clinical trial programmes and was driven by limited treatment options for RDs.

### Trust in relationships

Trust between doctor and patient was perceived to impact AE reporting. Participants described instances of miscommunication by patients in medical history taking. They also explained how patients sometimes purposefully withhold information for fear of judgement by MPs. Globally, the doctor-patient dynamic has shifted toward patient self-determination (Shutzberg 2021:233–234). However, in the account of one participant, paternalism still persisted in rural SA and limited doctor–patient dialogue. An unbalanced power dynamic in Sub-Saharan Africa has been described elsewhere in the literature (Camara et al. 2020:8). Patients’ selective sharing of medical history complicates MPs’ assessment of AE causality, jeopardising appropriate reporting (Hohl et al. 2018:4–6). Uncertainty regarding causality is a barrier to AE reporting for MPs (García-Abeijon et al. 2023:627; Hohl et al. 2018:6). Therefore, patient empowerment and effective communication are important facilitators. Involving patients actively in their care, including AE reporting, may improve safety data surveillance (Paola & Claudio 2020:3). Participants felt that PAOs could assist in levelling the perceived power imbalance by educating and empowering patients (Müller, Rach & Salloch 2021:2). In contrast, their direct involvement in the AE reporting process was not supported because of potential conflicts of interest, such as pharmaceutical industry funding of PAOs (Rose et al. 2017:349). However, conflicts of interest can be mitigated or overcome through robust governance mechanisms such as ethical oversight (Ehrlich et al. 2019:6).

In contrast, participants consistently demonstrated trust in pharmacists for AE reporting, which is aligned with the pharmacist scope of practice and which reflects pharmacists’ expertise in pharmacodynamics and the pharmacological mechanisms of AEs (South African Pharmacy Council [SAPC] 2017:32, 91). The participants’ value of pharmacists aligns with reported feedback from North American MPs. For example, in a qualitative study, a Canadian MP stated: ‘I don’t have time to take ownership [of routine reporting], but I would happily collaborate with the pharmacists and do my part in it’ (Hohl et al. 2018:8). A collaborative position was similarly supported by the South African participants in this study. As a pharmacist, the primary researcher (LK) acknowledged pharmacists’ roles in AE reporting and management. However, consistent with LK’s experience, South African pharmacists also face resource challenges (Luvhimbi et al. 2026:5). Therefore, alternative interventions for improving AE reporting rates are needed. The role of pharmacy support personnel in the AE reporting process should be considered. Interviewed MPs cited lacking administrative support as a barrier to AE reporting. For pharmacist reporting, administrative burdens could be carried by post-basic pharmacists’ assistants or pharmacy technicians, with pharmacist oversight.

### Systemic trust

Regulatory authorities stipulate that all AEs are reported (SAHPRA 2025:12–13). In contrast, all except one participant preferred to report only serious, unexpected or causally confirmed AEs, which is consistent with the broader literature (García-Abeijon et al. 2023:627; Hohl et al. 2018:6). The one participant who believed that all AEs should be reported had significant experience on pharmacy and therapeutics committees (PTCs), to which this view was self-attributed. This participant described AE reporting via the pharmacy and case assessment at local and provincial PTCs. Although an onerous process, this was seen to provide robust safety oversight. The perception that all AEs should be reported, in the context of significant PTC involvement, indicates that awareness of the significance and purpose of AE reporting impacts reporting rates. Participants described engaging in the pharmacovigilance system at their own cost, with several indicating uncertainty regarding its processes and perceived benefits. Inclusion of pharmacovigilance into medical school curricula was suggested by a participant and is a recommendation made as an outcome of this research. As key sources of safety data, educating MPs on pharmacovigilance systems may encourage their participation.

Participants hesitated to report isolated or rare AEs and opted to only report AEs they felt were justified. This reveals uncertainty, or an underlying mistrust in pharmacovigilance, a known contributor to underreporting (Güner & Ekmekci 2019:18–19). In pharmacovigilance practice, aggregated data is analysed to establish true risk-benefit profiles of medicines (SAHPRA 2022:5, 9, 2026:19–20). Global data aggregation is especially relevant in RDs because of small patient populations. Reporting these AEs is important, since each case, even rare or isolated events, contributes significantly to the global dataset.

### Rationed resources constrain adverse event reporting and patient care

Rationed resources featured consistently throughout the interviews, specifically: inadequate resources placing limits on AE reporting, and limited resources for patient care. Healthcare systems face resource constraints across the globe, most significantly in developing nations (Adachi et al. 2023:6–7, 17). This is especially pertinent to RDs, which can be expensive to treat (Kwaan et al. 2026:5). Participants described their struggle with the obligation to provide optimal patient care using limited resources, providing further insight into AE reporting barriers in the RD context. Decisions in relation to resource constraints revealed both ethical and systemic tensions around the reporting process.

### Limited resources for reporting adverse events

Participants noted that many new products are registered in SA several years after global launch. Delayed launch in developing nations, including SA, is attributed to limited commercial viability and slow regulatory processes (Narsai, Williams & Mantel-Teeuwisse 2012:33). This seems to demotivate MPs to report AEs because they perceive that their contribution would be insignificant, considering the substantial post-market data already collected. Global literature confirms that this perception discourages AE reporting, as does the conviction that safety profiles of marketed medicines are already known (García-Abeijon et al. 2023:627).

However, participants recognised the value of AE reporting for generating real-world data in African patients. They noted the limited evidence regarding RD genetic variants and phenotypes in these populations. There is vast genetic diversity in Africa (Lumaka et al. 2022:3), with South Africans being highly genetically diverse, reflecting the regional history of population mobility (Lankheet et al. 2025:2, 8; Sengupta et al. 2021:2). Except for infectious diseases, few clinical trials are undertaken in Africa as a result of high costs, complexity and legal hurdles (Mutebi et al. 2020:943). As a result, African RD patients are often underrepresented in pre-registration clinical trials, heightening the importance of safety surveillance for these populations. South African MPs routinely prescribe medicine with limited knowledge of its specific effects in their own patients, particularly in treating RDs. A facilitator of their AE reporting may be improved feedback regarding their report. Defined, standardised mechanisms are needed for responding to a reporter, explaining how their data contributed to knowledge of the medicine’s safety in the local patient population. This would generate trust in pharmacovigilance systems and may improve reporting rates.

### Limited resources for patient care

A significant identified barrier to AE reporting in RDs is limited resources for patient care. Participants repeatedly returned to this topic. Underreporting of AEs is widespread (Pal et al. 2013:79), but pervades practice in resource-limited settings like sub-Saharan Africa (Aagaard et al. 2012:1175–1177; Bogolubova et al. 2018:1). In RDs, this is compounded by limited treatment options, which discouraged this study’s participants from reporting AEs because of a perceived pressure to preserve available therapies. Medical practitioners’ reluctance to report AEs for fear of withdrawal of therapies from the market reflects their sense of medicine stewardship, consistent with a ‘physician-in-the-lead’ approach (Moleman et al. 2022:233). This reluctance also reveals a justifiable insecurity in the medicine supply chain and the performance of institutional authorities to ensure treatment access. It highlights the limitations of the broader healthcare system, especially in providing care for RDs. Their reluctance stems from the ethical dilemmas around treatment preservation versus AE reporting. If more treatment options were available, MPs might report AEs more freely.

The participants’ sense of stewardship of treatment resources caused them concern related to direct patient reporting of AEs to regulatory authorities or medicine manufacturers. Direct patient reporting may improve post-market surveillance and generate meaningful safety information (Paola & Claudio 2020:1, 3). While most participants acknowledged patient autonomy and their right to report AEs, they were concerned about the accuracy of reported data. They questioned patients’ ability to determine which clinical events were relevant to report as AEs. They also doubted patients’ ability to effectively report AEs in the context of polypharmacy, which is known to be associated with AEs, including drug interactions (Phoka & Khunou 2026:1, 3). Their concern was for the impact of irrelevant or erroneous AE reports upon medicine safety profiles. Regulators employ quality assurance procedures on AE datasets (European Medicines Agency [EMA] 2020:2); their processes ensure the quality of data entered but cannot directly verify the accuracy of information provided by the reporter.

It is this accuracy that raised concern regarding reports submitted directly by patients. The impact of singular, potentially irrelevant reports can be mitigated through several pharmacovigilance activities, such as: (1) advanced computing techniques and data mining (Ventola 2018:340, 349), (2) verification of safety signals to establish AE causality using multiple data sources (Ventola 2018:350) and (3) medical review of AEs by medicine manufacturers prior to regulatory submission. Pharmacovigilance analyses conducted on global AE data are highly sophisticated and can detect safety signals that are not apparent in isolated datasets (Brand et al. 2026:616–617, 625). This reinforces the importance of contribution to global AE data, especially rare AEs or those associated with RDs.

### Adverse event reporting as an aspect of risk management

The participants displayed a high degree of risk tolerance for treatment gain, especially in paediatric and RD settings. This tolerance was anchored in their perceptions regarding the risk–benefit balance of medicines, and it influenced their clinical practice, including AE reporting.

### Perceptions of the risk–benefit balance of medicines

The risk–benefit balance of a medicine involves the evaluation of its positive therapeutic effects in relation to its risks to patients or public health, relying on reported AEs to characterise risks (SAHPRA 2026:8–9, 19–20). Participants validated the importance of AE reporting based on their reliance on accurate medicines information to assess patient risk and support prescribing. Ongoing safety monitoring of medicines allows manufacturers to fully characterise their risk profiles in the post-market setting (SAHPRA 2022:7, 2026:12). Participants also noted the value of safety data collection for positioning medicines in terms of lifelong patient benefit.

### Impact on clinical practice

The risks incurred by MPs in their prescribing practice were discussed by several participants. This was meaningful to them, especially in treating RDs, in which the condition and/or medicines may be unfamiliar. They reduced this risk through collegial support and by actively involving their patients in healthcare decisions. Evidence-based medicine involves patients participating in treatment decision-making, accounting for their values together with research evidence and MPs’ expertise (Triposkiadis & Brutsaert 2025:2). Participants indicated that they rely on information regarding medicine safety to inform patients of risks and include them in treatment decisions. Adverse event reporting is the primary means of post-market safety surveillance (Pal et al. 2013:75). It thus provides information that empowers MPs and patients to participate in evidence-based medicine. Participants described how they may decide to treat through AEs, even serious ones, for the life-saving benefit of singular RD treatment options. This can confound their decision about whether to report an AE while continuing treatment using the implicated medicine. This presents an ethical dilemma between patient benefit and harm, which likely contributes to their reluctance to report AEs. Patient advocacy organisations were highlighted here for patient support, which is aligned with their key values (Müller et al. 2021:2, 7). Patient advocacy organisations were perceived by participants to offer more value in this role than in any direct involvement in the AE reporting process.

Medical practitioners also incur risk through off-label medicine use. They must justify their prescribing decisions outside the scope of regulatory-approved indications. Participants’ obligation to provide patient care seemed to drive their risk tolerance in this regard, with off-label use cited as a means of accessing treatment in paediatric and RD settings. The literature cites medicine repurposing as a valuable opportunity to address unmet therapeutic needs in RDs (Jonker et al. 2024:1). Repurposing is the development of existing medicines beyond the scope of their registration to obtain new regulatory-approved indications (Jonker et al. 2024:1). This is important in RDs, as most lack registered treatments. This is because their development is hindered by small patient populations, disease complexity, unclear pathology and high cost (Jonker et al. 2024:1). Repurposing leverages existing knowledge of medicines to improve development timelines. Off-label use of medicines generates valuable information regarding safety and efficacy, which can contribute to repurposing. However, this value is limited to information that is documented, including reported AEs.

### Strengths and limitations

The strengths of this study include:

- The qualitative methods elicited meaningful and nuanced insights into MPs’ perceptions and the issues underlying AE reporting behaviour, with valuable ideas on how to improve AE reporting in the South African or similar settings.
- Qualitative methods provided depth to the data which might not have been possible with survey or structured interview methods. Credibility and depth of analysis were enhanced by reflexive awareness in the process of data analysis.
- Individual interviews were facilitated by a pharmacist, which provided privacy and improved the credibility of the process. Focus groups facilitated by interviewers without a healthcare background would have been less effective for this kind of sample.

Limitations include:

- Potentially limited transferability of the results beyond the South African urban setting was noted. However, most participants held highly specialised medical qualifications and both local and international collaboration among such experts is common. Therefore, their perceptions are expected to align with those of MPs in other settings. This is especially relevant to resource constraints for RD patient care (including AE reporting), and to their limited understanding of pharmacovigilance systems.
- Several of the challenges in managing RDs may be applicable to all settings. However, some of these challenges, such as resource constraints, may apply more specifically to relatively similar contexts, such as other African countries.

### Recommendations

This research indicates that interventions to improve AE reporting by MPs should: (1) target improved medicine supply to expand treatment options, especially for RDs, thus reducing MPs reluctance to report AEs because of concern about losing treatment options; (2) improve feedback to reporters of AEs; and (3) raise awareness of AE reporting by educating MPs to understand medicine safety surveillance systems, with inclusion of pharmacovigilance into medical curricula.

Looking beyond MPs, the role of pharmacy support personnel in administering AE reporting with pharmacist oversight should be explored.

Further research into the role of PAOs in supporting patients to effectively engage with MPs regarding their experiences of AEs is recommended. They may offer insight regarding practical solutions for enhancing AE reporting by MPs, clinical management of AEs, patient communication regarding AEs, and increasing awareness of AEs. Finally, research on RD patients’ perspectives on AE reporting would add value to the conversation regarding direct patient reporting, which would supplement this research on MPs’ perspectives.

## Conclusion

This study revealed important insights into the significance and meaningfulness of barriers that MPs face with respect to AE reporting relevant to RDs. The generated themes reflected the experience which underpinned their perceptions. Trust relationships were central to their clinical decision-making, including AE reporting, with evidence that supportive structures related to medical practice and treatment access may improve their likelihood of reporting. Limited treatment access was a primary concern for participants. This demonstrated the interrelatedness of aspects of the healthcare system and the negative impact of supply chain issues on pharmacovigilance practices. Perceived risk was a key feature of these MPs’ practice, impacting their perceptions regarding AE reporting. The MPs interviewed in this study demonstrated great respect for patients and subsequently held a principled belief in the value of AE reporting for the good of patients, despite their hesitancy with respect to its practical application.

Addressing systemic barriers to care, particularly resource constraints and supply chain issues, may promote willingness among MPs to report AEs. This would better inform pharmacovigilance systems that are designed to protect patients, including those living with an RD.
